# Neuroendocrine prostate cancer (NEPC) in focus: state of the art and future prospectives

**DOI:** 10.1007/s12672-026-04482-7

**Published:** 2026-02-14

**Authors:** Emilio Francesco Giunta, Giuseppe Schepisi, Sara Bleve, Riccardo Serra, Nicole Brighi, Irene Torresan, Elisa Tassinari, Sofia Zanuccoli, Cristian Lolli

**Affiliations:** 1 Department of Oncology, Istituto Romagnolo per lo Studio dei Tumori “Dino Amadori” (IRST) IRCCS, Via P. Maroncelli, 40, I-47014 Meldola, Forlì-Cesena Italy; 2 Medical Oncology, University Hospital and University of Cagliari, University Hospital “Duilio Casula”, Monserrato, Cagliari Italy; 3 Section of Innovation Biomedicine–Oncology Area, Department of Engineering for Innovation Medicine, University of Verona and University and Hospital Trust (AOUI) of Verona, Verona, Italy

## Abstract

Neuroendocrine Prostate Cancer (NEPC) is a rare and clinically aggressive subtype of Prostate Cancer (PCa) with its own biological behavior, unfavorable prognosis, and resistance to androgen receptor (AR) directed therapies. De novo NEPC account for less than 1% of PCa cases, whereas a later evolution from castration-resistant PCa (CRPC) to NEPC is more frequent (approximately 15–20% of cases), because of tumor lineage plasticity. The loss of AR signaling, low prostate-specific antigen (PSA) levels, visceral metastases, and significantly reduced survival are the main characteristics of this tumor subtype. In the last decade, the incidence of NEPC has increased, in conjunction with the increasingly frequent use of new AR pathway inhibitors (ARPIs) such as abiraterone, enzalutamide, apalutamide and darolutamide. The increasing incidence of NEPC and especially its disproportionate contribution to mortality in advanced PCa justify the clinical relevance and the growing scientific interest regarding this tumor subtype. By integrating emerging data on NEPC biology, diagnostics, and therapeutics, this review aims to provide a critical and forward-looking description of the current landscape.

## Key points

1. Neuroendocrine prostate cancer (NEPC) represents a rare, yet clinically aggressive subtype of prostate cancer (PCa) characterized by a distinct biological behavior, poor prognosis, and limited therapeutic options due to its resistance to androgen receptor (AR) directed therapies.

2. De novo aggressive variants are quite rare (less than 1% of cases), whereas a later evolution from CRPC to NEPC is more frequent, accounting for approximately 15–20% of cases.

3. By integrating emerging data on NEPC biology, diagnostics, and therapeutics, this review aims to provide a critical and forward-looking description of the current landscape.

## Introduction

Prostate cancer (PCa) is the most frequently diagnosed non-cutaneous malignancy among men in the United States and is a leading contributor to cancer-related mortality, ranking second only to lung cancer in terms of deaths among male patients [1].

Neuroendocrine PCa (NEPC) represents a rare, yet clinically aggressive subtype of PCa characterized by a distinct biological behavior, poor prognosis, and limited therapeutic options due to its resistance to androgen receptor (AR) directed therapies [2].

In a subset of patients, resistance to AR–targeted therapy is associated with the development of a distinct histologic phenotype that shares morphologic features with de novo small-cell PCa, a highly aggressive variant that accounts for less than 1% of cases at initial diagnosis [3].

In fact, over the course of disease progression, approximately 15–20% of patients with castration-resistant PCa (CRPC), will evolve into a phenotype that no longer relies on AR signaling for growth and survival [4, 5].

This transformation reflects tumor lineage plasticity and is associated with loss of AR signaling, low prostate-specific antigen (PSA) levels, visceral metastases, and significantly shortened survival [6, 7].

Although traditionally considered uncommon, the incidence of NEPC has been increasing, particularly in the context of treatment-emergent transformation following androgen deprivation therapy (ADT) and AR pathway inhibitors (ARPIs). It has been demonstrated that the incidence of PC has increased in the last 15 years compared to the fifteen years preceding the approval of ARPIs: in particular, between 1997 and 2011, the vast majority (over 85% of cases) of PCa patients expressed the AR, while NEPCs were rare (about 6%) and even rarer were the so-called AR−/NE − or “double negative” PCa (DNPCa, less than 5% of cases). After 2011, the incidence of NEPC tumors increased to about 13%, and DNPC cases are even more frequently found (over 20%) [6].

The rising clinical relevance of NEPC stems not only from its growing incidence due to selective pressure imposed by AR-targeted therapies administered in early settings (e.g. non-metastatic CRPC, metastatic hormone sensitive PCa-mHSPC), but also from its disproportionate contribution to mortality in advanced PCa [8].

By integrating emerging data on NEPC biology, diagnostics, and therapeutics, this review aims to provide a critical and forward-looking description of the current landscape. The goal is to identify gaps in knowledge, highlight opportunities for translational research, and inform the design of future clinical trials.

## Biological characteristics of NEPC

### Histopathological and morphological features

In recent years, our understanding of NE differentiation (NED) in PCa has evolved considerably, largely due to new clinical and molecular insights especially in tumors that progress under new generation androgen-directed treatments. This body of knowledge has led to substantial refinements in how NEPCs are defined and classified.

The Prostate Cancer Foundation (PCF) in 2013 first outlined a broad clinical and morphological framework capturing the full continuum of NE features in PCa. More recently, the World Health Organization (WHO) 5th edition (2022) introduced a more standardized classification that integrates histological and molecular criteria in line with approaches applied across organ systems [9].

Both the PCF 2013 and WHO 2022 frameworks define a core group of NE neoplasms as distinct tumor entities:

- Carcinoid tumor/well-differentiated NE tumor (NET): Rare, indolent tumors with uniform cells, low mitotic activity, and a characteristic nested or trabecular pattern. These lesions express classic NE markers—chromogranin A, synaptophysin, INSM1—but are negative for PSA and AR [10].
- Small cell NE carcinoma (SCNEC): An aggressive, poorly differentiated tumor resembling its pulmonary counterpart, marked by nuclear molding, high mitotic rates, necrosis, and minimal cytoplasm. SCNEC is commonly characterized by alterations in RB1, TP53, and PTEN and is linked to a poor clinical outcome [11].
- Large cell NE carcinoma (LCNEC): A less common variant with large polygonal cells, prominent nucleoli, high proliferative index, and NE marker expression, often clinically similar to SCNEC [12].
- Mixed NE–non-NE carcinoma (MiNEN): Tumors containing both NE (small or large cell) and conventional acinar adenocarcinoma elements, often arising in advanced or treatment-resistant settings [13].

Although both classification systems align on the main categories, the PCF 2013 further delineates morphologic patterns not formally recognized as distinct entities in the WHO 2022, including conventional adenocarcinoma with scattered NE cells identified by immunohistochemistry and Paneth cell–like NED characterized by eosinophilic cytoplasmic granules. WHO 2022 instead includes these within the spectrum of usual adenocarcinoma.

Conversely, the latest WHO 2022 framework introduces important updates. One is the definition of treatment-related NEPC (t-NEPC), a high-grade NE carcinoma that emerges under selective pressure from ADT or ARPIs. This entity typically shows RB1 and TP53 loss, absent AR expression, and reflects lineage plasticity and treatment-driven transdifferentiation. The WHO 2022 classification also underscores the role of molecular markers (e.g., RB1, TP53, AR, PTEN) and proliferation indices (mitotic count, Ki-67) to support diagnosis and prognostication. Another innovation is the consolidation of genitourinary NE tumors in a dedicated chapter, although t-NEPC remains within the prostate section due to its unique clinical context.

Despite these advances, certain interpretive challenges persist. For example, the presence of scattered NE marker-positive cells within conventional PCas—detectable via immunostaining for chromogranin or synaptophysin—is common, yet their prognostic value remains unclear, with conflicting data regarding their impact on outcomes [14]. Their sparse distribution further complicates accurate assessments, particularly in limited biopsy samples.

Paneth cell–like differentiation is another histologic variation characterized by eosinophilic NE cells resembling intestinal Paneth cells but lacking lysozyme [13]. Seen across tumor grades and sometimes exaggerated post-ADT, this pattern can mimic high-grade Gleason patterns, although limited evidence suggests a less aggressive behavior [13].

Historically, the term “prostatic carcinoid” described tumors with NE features and uniform architecture. However, most cases previously labeled as carcinoid are now understood to be conventional adenocarcinomas with focal NED [15]. True primary prostatic carcinoids—similarly to those originating in other organs—are exceedingly rare and should only be diagnosed when PSA expression and acinar morphology are absent.

High-grade NE carcinomas, including small cell and large cell variants, represent a small proportion of PCas but pose significant diagnostic challenges, especially as they frequently arise following ADT and may coexist with conventional adenocarcinoma, reflecting transdifferentiation [11, 16]. Shared molecular alterations such as TP53 mutations and RB1 loss support a common clonal origin. Large cell variants can resemble Gleason pattern 5 adenocarcinoma, complicating histological distinction. Typically, these tumors lack AR expression but may show focal positivity along with variable PSA staining.

Differentiating small cell PCa from metastases of NE tumors from other sites (e.g., lung or bladder) can be complex, particularly in androgen-independent cases where traditional immunomarkers are inconclusive. Here, molecular assays, such as detection of TMPRSS2-ERG fusions, can help confirm a prostatic origin [17].

Recent clinical trials have expanded eligibility to include patients with an aggressive variant PCa molecular signature (AVPC-MS), characterized by concurrent alterations in at least two of TP53, RB1, or PTEN [18]. To explore the association between NEPC and AVPC-MS, a retrospective analysis of 308 NEPC cases from the Tempus Lens dataset was performed [19]. Approximately 35% of patients met AVPC-MS criteria, while the remainder lacked the requisite combination of alterations. Demographics and survival outcomes were comparable between groups; however, AVPC-MS tumors demonstrated a significantly higher prevalence of TMPRSS2 and PIK3CA mutations, whereas other alterations such as BRCA2 and FOXA1 did not differ. These findings indicate that although a molecular overlap exists, NEPC and AVPC-MS frequently represent distinct biological entities and underscore the need for histopathological confirmation of NEPC, as relying solely on genomic signatures may not accurately capture its phenotype [19].

Finally, a distinct subset referred to as “PCa with diffuse NED” exhibits extensive NE marker expression but retains defining features of PCa, such as AR, PSA, and NKX3.1 positivity [20]. These tumors, often emerging after ADT, display a wide morphologic range from carcinoid-like to solid growth lacking gland formation. Absence of necrosis and peripheral palisading can help distinguish them from classic LCNEC [21]. Given these overlaps, such ambiguous cases are increasingly described using emerging terms like “amphicrine carcinoma” or “PCa with mixed small cell and acinar features,” underscoring the diagnostic complexity of this evolving spectrum [22].

### Molecular pathogenesis and cellular origin

**Fig. 1:**
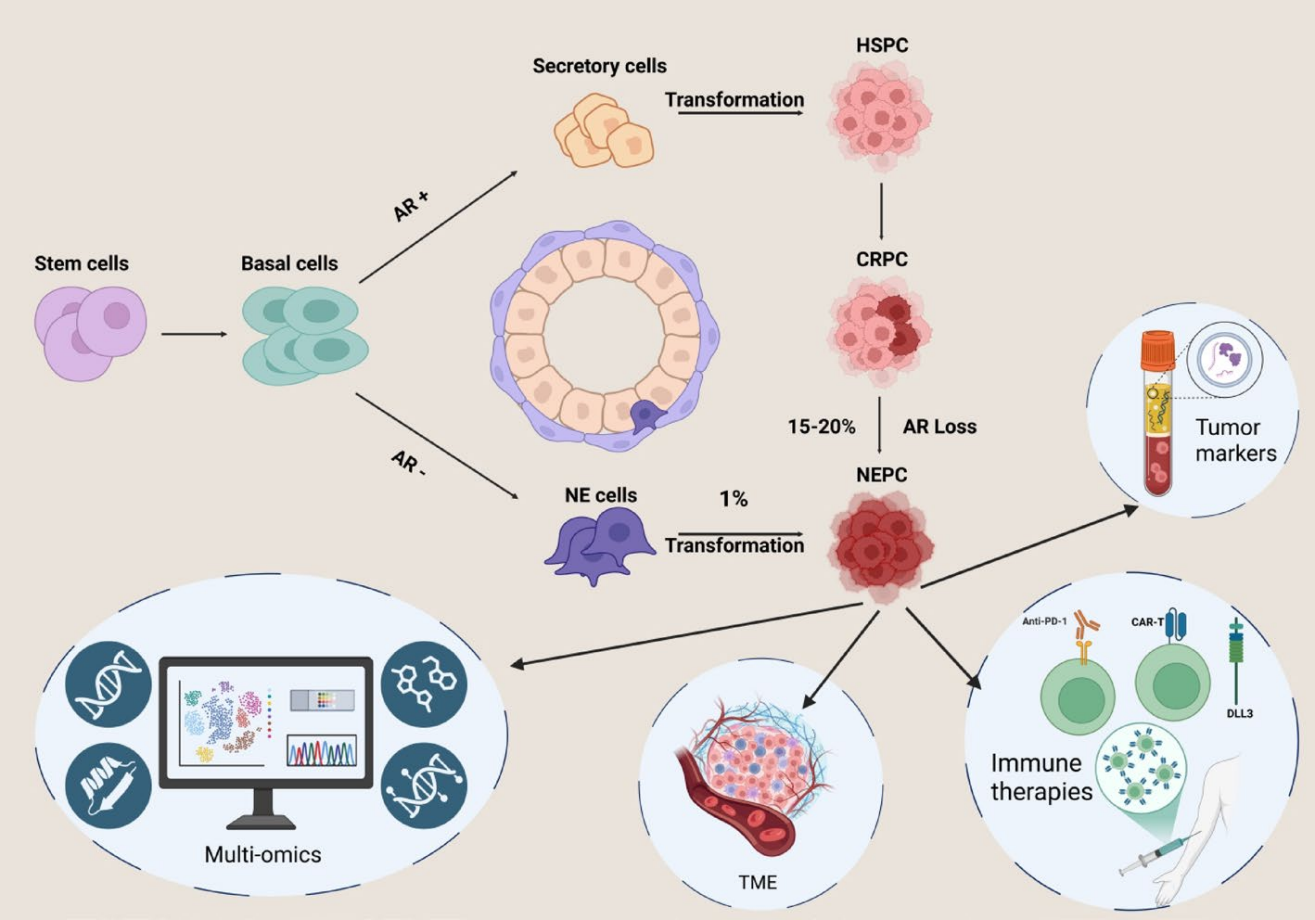
Tumorigenesis of NEPC, therapies and future prospectives. Abbreviations: AR: Androgen Receptor; CRPC: Castration-Resistant PCa; HSPC: Hormone-Sensitive PCa; NE: Neuroendocrine; NEPC: Neuroendocrine PCa; TME. Created in BioRender. **schepisi**,** g. (2025)** https://BioRender.com/onote06

The cellular origin of NEPC remains an area of active investigation, with two main hypotheses proposed: the NE cell origin model and the lineage plasticity model [16]. These frameworks seek to explain how NEPC develops, especially in the context of conventional PCa and its response to treatment. Figure 1.

The first hypothesis suggests that NEPC arises directly from resident NE cells within the normal prostate epithelium [23]. Although these cells constitute only a small fraction (approximately 1%) of the epithelial compartment, they play important paracrine roles by secreting bioactive peptides and hormones such as bombesin, serotonin, calcitonin, and parathyroid hormone–related peptide [24, 25]. Both normal NE and NEPC cells share expression of key markers including chromogranin A (CHGA), synaptophysin (SYP), and gamma-enolase (ENO2). Support for this model comes from genetically engineered mouse models, where targeted expression of the SV40 T antigen in prostate NE cells driven by the cryptdin-2 promoter resulted in proliferative NE lesions that progressed to metastatic, castration-resistant NEPC [26]. Despite these insights, the extent to which this pathway reflects human disease is uncertain, as mounting evidence favors an alternative mechanism centered on transdifferentiation.

The lineage plasticity model, now widely accepted, proposes that NEPC predominantly emerges through transdifferentiation of luminal adenocarcinoma cells, especially under selective pressure from AR pathway inhibition [5]. Molecular analyses have demonstrated a clonal relationship between adenocarcinoma and NEPC components, evidenced by shared genomic alterations such as TMPRSS2–ERG fusions, TP53 mutations, and RB1 loss [27].

Single-cell transcriptomic studies further corroborate this concept, showing that NE-like tumor cells often maintain luminal gene expression profiles rather than originating from basal or pre-existing NE cells [5]. These findings suggest that certain luminal adenocarcinoma cells can undergo lineage reprogramming in response to therapeutic pressure.

Genomic inactivation of RB1 and TP53 has emerged as a fundamental driver of this process [27–29]. Experimental models and patient samples reveal that their combined loss promotes NED, therapy resistance, and metastasis. Their inactivation contributes to increasing the so-called “lineage plasticity”, and is enabled by SOX2 hyperactivation: Mu et al. have demonstrated that SOX2 levels are significantly higher in NEPC versus adenocarcinoma, consistent with the higher incidence of TP53 and RB1 alteration in the first subtype (74 versus 39%, respectively) [28]. Based on murine models, McAuley et al. recently confirmed the role of SOX2 + prostatic luminal cells in cell survival and castration resistance mechanisms [29].

However, these genetic events alone are insufficient for full lineage switching, indicating the involvement of additional epigenetic and transcriptional regulators.

Key cooperating factors include *MYCN* amplification, which frequently co-occurs with aurora kinase A (*AURKA)* amplification, leading to N-Myc stabilization and promoting aggressive tumor growth [30]. *MYCN* has also been shown to interact with components of the Polycomb Repressive Complex 2 (PRC2), including EZH2 (Enhancer of Zeste Homolog 2), thereby suppressing AR signaling and upregulating genes involved in epithelial–mesenchymal transition [31]. Subsequent studies have validated *EZH2* as a critical epigenetic driver of NED in organoid models and have demonstrated its high expression in de novo NEPC patient tissue [32, 33].

A variety of complex mechanisms—including epigenetic alterations, non-coding RNAs, and dynamic transcriptional networks—contribute to the emergence and stabilization of the NE phenotype. These processes often act in concert with genetic disruptions and therapy-induced cellular plasticity, ultimately promoting lineage reprogramming in advanced PCa [34].

### Tumor microenvironment (TME)

While considerable research has elucidated the genomic and epigenomic drivers of NED from adenocarcinoma, there is growing recognition of the crucial role played by the TME in supporting this process [35]. The TME — including blood vessels, immune cells, fibroblasts, and the extracellular matrix — actively shapes NEPC development, progression, and metastatic spread.

ADT has been shown to remodel the stromal landscape in ways that favor NED [36]. Cancer-associated fibroblasts (CAFs) — a key component of the reactive stroma — are increasingly implicated in driving this transition [37]. Recent findings highlight a subset of fibroblasts marked by CD105 expression, which is associated with Epithelial-mesenchymal transition [38]. These CD105-positive fibroblasts can upregulate SFRP1, a factor that promotes NE gene expression [38]. Notably, treatment with enzalutamide increases CD105 levels, further linking AR-targeted therapy with TME changes that promote NEPC. Fibroblast activation protein (FAP), another CAF marker, has also been correlated with poorer outcomes and increased NE pathway activation [39]. Consequently, FAP-directed imaging and therapeutic strategies are being actively explored for NEPC management.

The TME in NEPC is less well defined but likely plays an equally important role in tumor evolution. Like other NE tumors, NEPC exhibits an immunosuppressive phenotype, characterized by features that enable immune evasion. ADT influences immune composition within the TME, enhancing the suppressive function of myeloid-derived suppressor cells (MDSCs) and affecting T-cell exhaustion and checkpoint pathways [40]. In preclinical settings, AR blockade has been shown to improve CD8 + T-cell function and increase sensitivity to PD-1 blockade [41].

Cytokine-mediated signaling further contributes to lineage plasticity. Interleukin-6 (IL-6) is a well-established driver of NE features in PCa cells, particularly under ADT pressure [42]. Other cytokines, such as IL-1β and IL-10, have been implicated in promoting skeletal metastasis and facilitating immune escape by upregulating PD-L1 expression [43]. Tumor-associated macrophages (TAMs), especially the M2-polarized subtype, are abundant within NEPC and support IL-6–mediated NE reprogramming. In addition, high mobility group box 1 (HMGB1) released in response to enzalutamide treatment recruits TAMs and sustains IL-6 signaling pathways [44].

Although immune checkpoint inhibitors have shown limited efficacy in unselected CRPC populations, the presence of PD-L1 expression in some NEPC cases suggests that immunotherapy could benefit selected patients [41]. However, the hypoxic microenvironment characteristic of NEPC may further limit immune cell infiltration and reduce antitumor immune responsiveness. Emerging preclinical evidence supports the potential of combining hypoxia-modulating agents with immunotherapies to overcome this barrier and enhance antitumor immunity in NEPC [4].

## Diagnosis of NEPC

### Clinical presentation and disease course

NEPC could be categorized based on the onset time: de novo and t-NEPC [45]. These two entities are different in terms of clinical presentation and disease course. De novo NEPC represents less than 2% of all PCa diagnosis, even if a focal NED can be found in a higher percentage of cases of localized disease [4, 29]. De novo NEPC is characterized by a higher prevalence of metastases at the time of diagnosis and a shorter OS if compared to t-NEPC [45]; however, these two entities share both an aggressive behavior and poor prognosis [46]. An autopsy series showed that up to 25% of mCRPC patients have signs of NED [47]. T-NEPC belongs to the so-called AVPC, which shows visceral metastases, lytic bone metastases, bulky tumor masses and low PSA values [45, 48]. In a multi-institutional prospective study, including 202 mCRPC patients, t-NEPC was found in less than 20% of the global cohort, mostly pretreated with ARPIs [5]. Clinical characteristics did not differ between t-NEPC and non-NE mCRPC, in terms of time from mCRPC to study entry, Gleason score at time of diagnosis, and sites of metastases. Among serum biomarkers, median serum NSE was higher in patients with treatment-emergent NEPC, whilst CGA and PSA were similar. The authors concluded that the overlap of the clinical features between treatment-emergent NEPC and non-NE mCRPC should call into question the recommendation for biopsy only for patients with aggressive phenotypic features [5].

A retrospective analysis of the SEER 17 registry, comprising 1,400 de novo and 143 treatment-emergent NEPC cases, found no major differences in baseline characteristics—including age, stage, and prior treatments—between the two groups. However, median OS was significantly shorter in t-NEPC (8 months) compared with de novo cases (11 months). In multivariable analysis, radiation therapy and surgery were associated with improved survival, whereas t-NEPC subtype, older age, and advanced disease stage predicted worse outcomes; in contrast, chemotherapy did not confer a significant survival benefit in either cohort [49].

### Imaging techniques

Conventional imaging techniques such as computed tomography (CT) and magnetic resonance (MR), are commonly used for PCa diagnosis and staging but they could have limited value in NEPC, apart from suggesting its onset in case of aggressive features (i.e., visceral metastases and lytic bone metastases) [50, 51].

Consequently, in the latest years clinicians have focused on new techniques for NEPC imaging.

The paradigm shift for imaging of NE tumors was achieved with the introduction of functional imaging modalities [52]; firstly, somatostatin receptor (SR) scintigraphy was adopted based on the well-known SR expression on the surface of well-differentiated NE cells [53]; later, the use of Positron Emission Tomography (PET) became the gold standard, with the use of 68Ga-somatostatin analogues [54]. It is important to underline the impact of these new techniques not only for a better disease staging but also for evaluating eligibility for somatostatin analogue and radionuclide therapy [55].

Concerning NEPC, to date, several PET tracers have been studied, even if to date none of them is recommended in clinical practice [50].

The 18 F-fluorodeoxyglucose (FDG) is a useful tracer for identifying tissues with high glucose metabolism. However, PCa often shows low glucose metabolism, and therefore its use is limited in classical adenocarcinoma histology [56]. Nonetheless, FDG could be a useful imaging technique for NEPC since high-grade NE cancer commonly shows a high glucose metabolism [57]. In detail, Spratt and colleagues demonstrated that FDG PET can detect visceral metastases in NEPC patients [57].

The 68Ga-somatostatin analogues, despite their potentiality, have not been extensively studied in NEPC. Some case series are available [58, 59], demonstrating their utility in this setting. However, more data is needed to fully elucidate the role of SR in NEPC, since some preclinical data suggests that the SR expression is inconstant in NEPC cell lines [60].

A new PET target in NEPC patients is delta-like ligand 3 (DLL3), an inhibitory notch ligand which is highly expressed on the surface of NEPC cells [61, 62], being also associated with poor survival [63]. Preclinical data showed the efficacy of [89Zr]Zr-DFO-SC16.56, composed of the anti-DLL3 antibody conjugated to DFO which chelate zirconium-89, in identifying NEPC cells [62]. The first promising results of a phase I/II study of immunoPET-CT imaging with [89Zr]Zr-DFO-SC16.56 have been recently published [64].

## Therapeutic approaches and emerging targets

### Standard treatment strategies: platinum-based chemotherapy

The therapeutic management of NEPC, particularly t-NEPC subtype, remains a major clinical challenge due to its inherent resistance to ARPIs and its aggressive clinical course [65, 66].

In the absence of NEPC-specific guidelines, treatment approaches are generally extrapolated from small-cell lung cancer (SCLC), given the morphologic and phenotypic similarities between the two entities.

To date, platinum-based chemotherapy remains the cornerstone of systemic therapy for NEPC [48]. Regimens typically consist of a platinum agent, most commonly either cisplatin or carboplatin, combined with etoposide, a topoisomerase II inhibitor. These patients may be treated with cytotoxic chemotherapy (i.e., cisplatin/etoposide, carboplatin/etoposide, docetaxel/carboplatin, cabazitaxel/carboplatin) with limited randomized data [67]. However, there is some recent evidence that treatment intensification (e.g. a combination of degarelix, docetaxel and darolutamide, as reported in a recent case report in one patient with de novo NEPC) could improve NEPC patient outcomes [68].

A phase II study by Aparicio et al. tested first line chemotherapy with carboplatin plus docetaxel (CD) with carboplatin, area under the curve (AUC) 5, plus docetaxel, 75 mg/m^2^ on day 1 every 3 weeks in a cohort of 120 NEPC patients. Second-line cisplatin plus etoposide (EP), with etoposide, 120 mg/m^2^, plus cisplatin, 25 mg/m^2^ was administered daily for 3 days every 3 weeks upon tumor progression [48]. The primary endpoints were to estimate the response rate and the time to progression for patients with NEPC treated with front-line CD and their response rate and time to progression to second-line EP following treatment with CD. After a median follow-up of 39.1 months (range, 1.07–62.47 months), 92.9% of patients experienced disease progression (PD) after CD, with a median time to PD of 5.1 months (95% confidence interval CI: 4.2–6.0.2.0 months). 70.5% of the patients received second-line EP on study, with a median time to PD of 3.0 months (95% CI: 1.6–3.5 months) [48].

A phase I-II trial by Corn et al. tested the association of carboplatin and cabazitaxel in a cohort of 160 mCRPC patients [69]. In the phase I the primary endpoint was to determine the maximum tolerated dose of carboplatin and cabazitaxel, while in the phase II the primary endpoint was PFS and patients were randomly assigned (1:1) centrally to intravenous cabazitaxel 25 mg/m^2^ with or without intravenous carboplatin AUC 4 mg/mL per min. At a median follow-up of 31.0 months (IQR 20.5–37.1), the combination improved the median PFS from 4.5 months (95% CI: 3.5–5.7 months) to 7.3 months (95% CI: 5.5–8.2 months; hazard ratio 0.69, 95% CI: 0.50–0.95, *p* = 0.018) [69].

### Limited efficacy of AR-directed therapies

As previously stated, the lack of efficacy of ARPIs in NEPC reflects a fundamental shift in tumor biology that renders these therapies ineffective. Unlike conventional PCa, which depends on AR signaling for proliferation and survival, NEPC exhibits a largely AR-independent phenotype, often accompanied by complete loss of AR protein expression or inactivity of downstream AR transcriptional programs. This biological divergence underpins the intrinsic resistance of NEPC to AR-directed strategies [30].

Given that NED may serve as a mechanism of resistance to standard ARPIs, patients with mCRPC displaying NE features have typically been excluded from clinical trials assessing the efficacy of ARPIs [69–72].

In a retrospective study by Farinea et al., 329 mCRPC patients were treated with abiraterone or enzalutamide as first, second or later lines; among these, 9.8% were NED. Across the study cohort, the presence of NED in mCRPC was consistently associated with worse clinical outcomes [74]. Patients with NED exhibited significantly shorter progression-free survival (PFS) compared to those without NE features (4.38 vs. 11.48 months; hazard ratio [HR] 2.51, 95% confidence interval [CI] 1.71–3.68; *p* < 0.05), as well as reduced disease control rates (DCR) and lower PSA response rates. When stratified by the line of therapy, this negative prognostic effect remained evident. In the first-line setting, mCRPC cases with NED showed a shorter median PFS of 8.5 months compared to 14.9 months in those without NED (HR 2.13, 95% CI 1.18–3.88; *p* < 0.05). Similarly, in patients receiving second-line or later therapies, NED was associated with a median PFS of 4.0 months versus 7.5 months (HR 2.43, 95% CI 1.45–4.05; *p* < 0.05), in addition to decreased DCR, diminished PSA response, and shorter overall survival (OS: 12.53 vs. 18.03 months; HR 1.86, 95% CI 1.12–3.10; *p* < 0.05). Notably, the detrimental effect of NED on PFS was consistent across clinical subgroups, and a trend toward worse outcomes was also observed among patients with a higher degree of NE involvement compared to those with more limited expression [74].

In a retrospective analysis, Xu and colleagues evaluated the clinical impact of NED on treatment outcomes in patients with mCRPC, using biopsy specimens obtained at the time of mCRPC diagnosis [75]. Among the 262 patients included in the study, NED was identified in 100 cases (38.2%), with 76 patients exhibiting a limited NE component (involving < 10% of tumor cells) and 24 displaying a more substantial component (≥ 10% of tumor cells). The presence of NED was significantly associated with reduced radiographic PFS (rPFS) in both treatment arms: patients receiving abiraterone had a median rPFS of 15.9 months compared to 19.5 months in those without NED (*p* = 0.010), while those treated with docetaxel demonstrated a median rPFS of 8.4 months versus 20.4 months (*p* = 0.016) [75].

### Novel molecular targets and early-phase clinical strategies

Recent advances in elucidating the molecular drivers of NEPC have identified several actionable targets, including AURKA, DLL3, immune checkpoints and other molecular targets [76]. The following section summarizes the most relevant targets for which completed and ongoing clinical trials are available, explicitly excluding data derived solely from preclinical studies (Table 1).

AURKA stabilizes N-Myc and prevents its proteasomal degradation in both human neuroblastoma and PCa [29, 76]. Through this interaction, N-Myc suppresses AR signaling and drives lineage plasticity, tumor aggressiveness, and AR-independent progression in preclinical models of PCa [77]. The AURKA catalytic inhibitor alisertib disrupts the AURKA–N-Myc complex, thereby attenuating N-Myc–driven signaling and inhibiting tumor growth. The phase II trial of the AURKA inhibitor alisertib in mCRPC with NE features did not meet its primary endpoint, reporting a six-month rPFS of 13.4% and a median OS of 9.5 months. However, a subset of exceptional responders achieved profound and durable benefit, including complete regression of visceral metastases, which correlated with molecular signatures of AURKA/MYCN activation [78].

DLL3 is an atypical Notch ligand markedly upregulated in NEPC and largely absent in adenocarcinoma [79]. Building on preclinical evidence, the bispecific DLL3/CD47 antibody peluntamig (PT217) is currently under evaluation in the phase I/II SKYBRIDGE trial (NCT05652686). This is a multi-cohort phase I/II dose escalation, dose expansion and combination clinical trial evaluating peluntamig (PT217), as a single agent or in combination with chemotherapy or immunotherapy, in DLL3 expressing NE carcinomas, including NEPC. Specifically, peluntamig is a bispecific antibody targeting both DLL3 and CD47. In one cohort of the trial, it is being administered either as monotherapy or in combination with atezolizumab in both frontline and relapsed NEPC; in another cohort, peluntamig is administered as a maintenance therapy following chemotherapy induction [80]. In December 2024, the FDA granted a fast-track designation to peluntamig for the treatment of t-NEPC [81].

NCT06941480 is a phase I trial, currently enrolling patients with SCLC and NEPC who progressed to first-line standard chemotherapy, in which ^177^Lu-DTPA-SC16.56 is administered. This drug is a Radioligand therapy (RLT) directed against DLL3, already studied in preclinical models [82]. Notably, patients undergo an ^89^Zr-DFO-SC16.56 PET/CT scan at screening to evaluate the in vivo distribution of DLL3.

In the context of RLTs, NCT06379217 is a phase I trial currently enrolling patients with metastatic NEPC randomized to receive a RLT selected on the basis of their predominantly expressed target evaluated at PET-CT among prostate specific membrane antigen (PSMA), Somatostatin Receptor 2 (SSTR2), and Gastrin Releasing Peptide Receptor (GRPR).

Complementing these targeted approaches, early-phase clinical trials are exploring rational combinations, including immune checkpoint inhibitors in both de novo NEPC and t-NEPC.

Clinical trials conducted in SCLC patients have highlighted the efficacy of combining anti-PD(L)1 drugs with chemotherapy in the first line setting [83]. In detail, atezolizumab or durvalumab – two anti-PD-L1 monoclonal antibody – combined with platinum-based chemotherapy and followed by immunotherapy maintenance are now the standard of care in SCLC patients without contraindications to immunotherapy [84, 85].

Therefore, the question arose whether NEPC behaved the same way as SCLC in terms of response to immunotherapy. Single agent immune checkpoint inhibitors are ineffective in NEPC patients, as shown in a phase II clinical trial with avelumab [86].

Recently, the bispecific T cell engager (BiTE) molecule tarlatamab, designed to bind DLL3 on target cancer cells and CD3 on T cells, was tested in some studies involving SCLC patients. In a phase 1b study (NCT04702737) the obtained results in the DLL3 expressing NEPC subgroup were interesting, with a manageable toxicity profile [87].

A retrospective analysis conducted by Bhinder and colleagues profiled the immunogenomic landscape of 230 patient biopsies, showing that NEPC have usually an immune-depleted TME – T-cell depletion and high PD-L1 expression, as compared to adenocarcinoma [88].

To date, few studies or case series have evaluated the combination of platinum-based chemotherapy and immune checkpoint inhibitors in NEPC patients, with discouraging results [88–90]. A phase 2 single-arm trial with a quadruplet - nivolumab, ipilimumab, carboplatin and cabazitaxel - for up to ten cycles followed by maintenance with nivolumab and ipilimumab is currently ongoing (NCT04709276).

A phase II single arm trial (DURVASCC) is currently evaluating the association of carboplatin/cisplatin, etoposide and durvalumab as first line treatment in patients with advanced extrapulmonary small cell cancer, thus including those arising in the prostate (NCT06464068).

The phase 1b/2 KEYNOTE-365 (cohort I) trial investigated pembrolizumab in combination with carboplatin and etoposide in patients with treatment-emergent and de novo metastatic NEPC. Among 40 randomized patients, the combination demonstrated higher objective response rates (33% vs. 6%), improved PSA responses (37% vs. 18%), and prolonged median rPFS (5.1 vs. 4.0 months) and OS (11.4 vs. 7.8 months) compared to chemotherapy alone, suggesting that adding pembrolizumab to platinum-based regimens may enhance efficacy without significant additional toxicity in NEPC [92].

The PLANE-PC study (NCT04848337) is a multicenter phase II clinical trial, which evaluated the combination of pembrolizumab and lenvatinib in 33 NEPC patients. 2 prior chemotherapy regimens were allowed. It is noteworthy that, in this trial, patients was enrolled also in case of aggressive variant features such as visceral metastases and a PSA < 5 ng/dL, RB1 deletions/mutations or trans/poorly-differentiated carcinoma. The combination demonstrated a PR in 3 patients (14%) and a SD in 7 patients (33%); moreover, 5 patients reached a 6 months of PFS, with a interesting safety profile [93].

NCT04926181 is an ongoing phase II single-arm trial evaluating the combination of cetrelimab (anti–PD-1) and apalutamide in mCRPC patients with histologic and/or genomic evidence of t-NEPC who have progressed after at least one prior ARPI. The rationale is based on the observation of persistent AR expression in the majority of t-NEPC biopsies, thus justifying the continuation of AR blockade and possibly increasing the efficacy of immunotherapy.

NCT03910660 is an open-label multicenter phase Ib/II clinical trial in mCRPC patients, aiming at the identification of the recommended dose and the evaluation of efficacy and safety of BXCL701 (talabostat), alone or in combination with pembrolizumab. BXCL701 is an orally administered, nonselective dipeptidyl peptidase inhibitor that has demonstrated the ability to enhance PD-1 blockade efficacy in preclinical models by modulating the tumor microenvironment (TME) [94, 95].

In the phase IIa of this trial, which included both de novo and t-NEPC, the combination of BXCL701 and pembrolizumab have demonstrated to be effective and safe [96]. Based on these results, the FDA has granted Fast Track Designation to BXCL701 for NEPC patients [97].

Novel immunotherapeutic strategies such as LeY-directed CAR T cells have also demonstrated potent, antigen-specific cytotoxicity in preclinical NEPC models, supporting further clinical development [98].

Additional insights into NEPC pathogenesis have identified further potential therapeutic targets, including enhancer of zeste homolog 2 (EZH2), lysine-specific demethylase 1 (LSD1), and poly(ADP-ribose) polymerase (PARP). Among these, EZH2 stands out as a central mediator of epigenetic reprogramming and transdifferentiation of PCa into NEPC, making it a particularly compelling candidate for targeted intervention [99]. Although multiple EZH2 inhibitors are in clinical development for mCRPC, their activity in NEPC has been demonstrated only in preclinical models, where EZH2 blockade reduced tumor invasiveness and enhanced sensitivity to AR pathway inhibition [98–101]. Similarly, LSD1 inhibition with agents such as bomedemstat has shown preclinical efficacy against NEPC independent of AR signaling, whereas TROP-2 and PARP-targeted approaches remain at an early stage and require further clinical validation [102–104].

**Table 1:** Clinical trials of novel targets and combination therapies in neuroendocrine PCa (NEPC)

Target	Drug/intervention	Trial (ID)	Phase	Population	Status
ATR	Topotecan versus topotecan + berzosertib (M6620)	NCT03896503	II	Advanced pulmonary and extrapulmonary small cell cancer, including NEPC	Active, not recruiting
AURKA	Alisertib	NCT01799278	II	mCRPC with NE features	Completed; primary endpoint not met
DLL3/CD47 (bispecific antibody)	Peluntamig monotherapy or in combination with platinum-based chemotherapy/atezolizumab	SKYBRIDGE (NCT05652686)	I/II	NEPC (de novo and t-NEPC), DLL3+	Recruiting
DLL3 (RLT)	^177^Lu-DTPA-SC16.56	NCT06941480	I	NEPC after first-line chemotherapy	Recruiting
PSMA, SSTR2 and GRPR Targeted Radioligand	^177^Lu-PSMA-617 ^177^Lu-DOTA-TATE ^177^Lu-NeoB	NCT06379217	I	Metastatic NEPC	Recruiting
Anti-PD-1	Pembrolizumab + carboplatin/etoposide	KEYNOTE-365	Ib/II	De novo and treatment-emergent NEPC	Completed
	Pembrolizumab + lenvatinib	PLANE-PC (NCT04848337)	II	NEPC; including AVPC features (PSA < 5 ng/dL, RB1 alterations, visceral disease)	Recruiting
	Pembrolizumab + etoposide/docetaxel/cisplatin/carboplatin	NCT03582475	I	advanced urothelial small cell cancer and NEPC	Completed
	Cetrelimab + apalutamide	NCT04926181	II	mCRPC with histologic/genomic t-NEPC post-ARPI	Recruiting
	BXCL701 (talabostat) ± pembrolizumab	NCT03910660	Ib/II	mCRPC (de novo and t-NEPC)	Recruiting
	Nivolumab + ipilimumab	NCT02834013	II	Rare tumors (including NEPC)	Active, not recruiting
	Nivolumab + ipilimumab	NCT03333616	II	Rare genitourinary tumors, including NEPC	Active, not recruiting
	Nivolumab + ipilimumab + cabozantinib	NCT03866382	II	Rare genitourinary tumors, including NEPC	Recruiting
	Nivolumab + ipilimumab + cabazitaxel + carboplatin	NCT04709276	II	NEPC; including AVPC features	Recruiting
	Vudalimab (XmAb20717)	NCT03517488	I	Advanced solid tumors (including NEPC)	Completed
Anti-PD-L1	Carboplatin/cisplatin, etoposide and durvalumab	DURVASCC (NCT06464068)	II	Advanced extrapulmonary small cell cancer, including NEPC	Recruiting
	Avelumab	NCT03179410	II	NEPC	Completed
DLL3 + BiTE	Tarlatamab	DeLLpro-300 (NCT04702737)	Ib	NEPC	Completed
DNMT	Guadecitabine (SGI-110) + pembrolizumab	NCT02998567	I	Advanced solid tumors (including NEPC)	Recruiting

Anti‑PD‑1: Programmed Death-1 checkpoint inhibitor; ARPI: Androgen Receptor Pathway Inhibitor; Nivolumab: Anti‑PD‑1 monoclonal antibody; ATR: ataxia telangiectasia and Rad3-related protein; AURKA: Aurora Kinase A; AVPC: Aggressive Variant PCa; mCRPC: Metastatic Castration-Resistant PCa; DLL3: Delta-Like Ligand 3; DNMT: DNA methyltransferase; ^177^Lu-DTPA-SC16.56: Radio-labeled antibody with Lutetium-177 chelated by DTPA targeting SC16.56 (DLL3); EZH2: Enhancer of Zeste Homolog 2; MEK/ERK: Mitogen-activated protein kinase kinase/Extracellular-signal-regulated kinase; NE: Neuroendocrine; NEPC: Neuroendocrine PCa; t‑NEPC: Treatment-Emergent Neuroendocrine PCa; PI3K/Akt/mTOR: Phosphoinositide 3-kinase, protein kinase B, mammalian target of rapamycin. PSA: Prostate-Specific Antigen; RB1: Retinoblastoma 1 gene; SRC: a nonreceptor tyrosine kinase family

### Biomarkers

The diagnosis of NEPC is presently based on the histopathological evaluation of tissue biopsy specimens. However, acquiring metastatic tissue—especially for repeated sampling—is often limited by procedural challenges, patient risk, and logistical barriers, making serial biopsies impractical in most cases. Despite this, longitudinal monitoring remains essential for detecting the emergence of NEPC features at an early stage.

A range of biomarkers is commonly used to identify NEPC (Table 2). The most extensively investigated markers comprise chromogranin A (CgA), synaptophysin, and neuron-specific enolase (NSE). NSE, while highly sensitive due to its broad expression in neuronal and NE cells, lacks sufficient specificity [107, 108]. Conversely, chromogranin A is considered more specific, as it constitutes a principal component of the dense-core secretory granules characteristic of NE cells [109]; however, its clinical utility is limited by the fact that circulating levels can also be affected by non-neoplastic conditions as well as by the use of certain medications or dietary factors.

Molecular profiling through genomic and epigenomic analyses has emerged as a more robust and clinically relevant strategy for detecting NE features and informing treatment. Comprehensive whole-exome, transcriptomic, and genome-wide methylation studies on metastatic tissue biopsies have confirmed that NE phenotypes in mCRPC frequently arise through divergent clonal evolution, characterized by RB1 and TP53 loss, profound epigenetic reprogramming, and the activation of distinct NE transcriptional programs associated with aggressive clinical behavior [5]. The development of integrated molecular classifiers based on these multiomics data has demonstrated high diagnostic accuracy and is presumed to be adaptable to liquid biopsy approaches, offering a promising avenue for earlier and less invasive detection as well as improved therapeutic stratification [34].

Building on these tissue-based insights, attention has increasingly shifted toward minimally invasive strategies to capture the same molecular signals with particular focus on DNA methylation–based approaches [110]. Among these, cfMeDIP-seq, which performs genome-wide methylation analysis on circulating cell-free DNA, has shown remarkable ability to discriminate NEPC from castration-resistant prostate adenocarcinoma (CR-PRAD). In a recent study, this technique achieved outstanding sensitivity and specificity, supporting methylation-based liquid biopsy as a promising and rapidly advancing strategy for noninvasive NEPC detection [111]. Another recent study has also leveraged cfDNA methylation analysis to distinguish NEPC from CRPC. Using a targeted methylation panel derived from metastatic tumor signatures, this work demonstrated high accuracy in identifying NEPC and estimating tumor burden from plasma samples, further supporting methylation-based liquid biopsy as a powerful approach for early and noninvasive detection of NE transformation [112]. Despite promising results, broader implementation of these tools is still hindered by limited assay availability, costs, and lack of standardization.

A recent study presented at ASCO Gu 2025 introduced cfChIP-seq as a noninvasive approach to track NEPC transformation in CRPC. By profiling H3K27ac marks on cfDNA, the assay generated NE-specific (NE-score) and AR-driven (AD-score) signals from plasma samples. In a small longitudinal cohort, rising NE-scores closely mirrored disease progression and, in some cases, preceded histologic confirmation, outperforming total tumor cfDNA, while AD-scores correlated with PSA levels and AD tumor burden [113].

Analysis of circulating tumor cells (CTCs) represents a complementary strategy to ctDNA profiling. The potential of CTC morphology as a noninvasive indicator of NEPC transformation was demonstrated in a study showing that CTCs from NEPC patients exhibit a distinct phenotype with smaller size, low AR and cytokeratin expression, and frequent CK−/AR − subpopulations. When applied to an independent cohort, the classifier identified NEPC-like CTCs in approximately 10% of CRPC cases, which were associated with high CTC burden and visceral metastases [114]. Brown et al. subsequently confirmed that NEPC phenotypes in CTCs are independently associated with inferior survival outcomes in patients with mCRPC undergoing ARSI therapy [115]. A clinical-grade multiplex RNA qPCR assay on CTCs for NEPC detection via liquid biopsy was validated in a prospective metastatic PCa cohort [116]. The assay demonstrated approximately 51% sensitivity and 91% specificity per sample, while incorporating serial sampling achieved 100% diagnostic accuracy on a per-patient basis. In the phase II clinical trials involving patients treated with ARSI considered by the authors, the presence of NEPC markers in CTCs, even alongside preserved AR target gene expression, was associated with adverse outcomes and early evidence of NED [114]. At ASCO Gu 2025, a CTC RNA sequencing approach was used to classify metastatic PCa into transcriptional phenotypes, identifying a NE subtype associated with poor overall survival (3.7 months). NE CTCs lacked AR alterations and were enriched for RB mutations. Integrating NE phenotype with high-risk genomic alterations further identified patients with the worst outcomes, highlighting its potential for prognostic stratification [117].

**Table 2:** Established and emerging Circulating biomarkers in NEPC

Biomarker	Specificity	Sensitivity	Advantages	Limitations
Chromogranin A (CgA)	Limited	Moderate	Cost-effective Widely accessible	Drug and dietary interference Variable correlation with disease status Heterogeneity of expression
Neuron-Specific Enolase (NSE)	Low	High	Cost-effective Widely accessible	Biological variability Limited prognostic power
cfDNA methylation (Cf-MEDIP-seq; NEMO assay)	High	High	Cf-Medip-seq: Subtype discrimination Genome-wide coverage Nemo assay: • Clinical scalability (cost and faster assay) • Tumor burden estimation • Correlation with outcomes	Dependence on tumor fraction Cf-medip seq: • Cost, • Technical complexity Nemo assay: • Subtype heterogeneity • Restricted genomic space
cfChIP-seq (NE-score/AD-score)	Potentially High (small sample size)	Potentially High (small sample size)	Detection of NE transformation before histologic confirmation	Technical complexity; limited resolution of mixed disease
CTCs (molecular profiling)	Variable	High when combined with serial testing and RNA profiling	Dynamic disease tracking; molecular and phenotypic resolution; correlation with clinical outcomes; early detection potential; applicability to multiomics	Intra-patient heterogeneity; no standardized molecular/protein signature; demands complex platforms with performance influenced by technical and pre-analytical factors

CgA: Chromogranin A; NSE: Neuron-Specific Enolase; cfDNA: Cell-free DNA; Cf-MEDIP-seq: Cell-free Methylated DNA Immunoprecipitation Sequencing; NEMO: Neuroendocrine Methylation assay for Oncology; cfChIP-seq: Cell-free Chromatin Immunoprecipitation Sequencing; NE-score/AD-score: Neuroendocrine/Adenocarcinoma transcriptional scores; CTCs: Circulating Tumor Cells

### Future perspectives and challenges

The early detection of lineage plasticity remains one of the major challenges in the management of treatment-emergent NEPC; building on the strategies described above, the development of non-invasive approaches, such as liquid biopsy–based assays and advanced imaging modalities, represents a critical step toward real-time disease monitoring and lays the groundwork for integrating multiomics data—including transcriptomics, metabolomics, lipidomics profiling, and radiomics—into precision medicine approaches for NEPC.

The emergence of single cell sequencing technologies has transformed the ability to profile gene expression and dissect intra-tumoral heterogeneity at an unprecedented resolution. By capturing the transcriptomic landscape of individual tumor cells, these approaches generate critical data that enhance our understanding of the molecular features, clonal architecture, and phenotypic diversity of NEPC [118].

At the same time, advanced machine learning approaches provide essential analytical frameworks to uncover meaningful biological insights embedded within large and complex datasets. These computational methods enable the identification of subtle patterns and associations that would otherwise remain undetected, thereby complementing multiomics and single-cell data in NEPC research [119].

In a study by Bian and collegues, an integrated multiomics framework, encompassing genomic, transcriptomic, epigenomic, proteomic, and metabolomic analyses, offers an unparalleled approach to decipher lineage plasticity and therapeutic resistance in NEPC [120]. Single-cell sequencing efforts within PCa cohorts have revealed subpopulations of NE cells characterized by RB1 and TP53 loss, MYC amplification, and unique chromatin states distinct from adenocarcinoma [120].

Building on this, multiomics classifiers such as the NEP100 model, developed by Shen and colleagues via random forests on integrated bulk and single-cell data, stratify NEPC into four biologically distinct subtypes, each associated with differential prognosis and potential therapeutic pathways (NEPup, NEPdown signatures) [121].

Additionally, integrated metabolomic and transcriptomic profiling of cell line models comparing adenocarcinoma and small-cell NE carcinoma has elucidated subtype specific metabolic signatures, revealing, for instance, enhanced glycolysis, increased lipid β-oxidation and reduced citrate accumulation in NEPC relative to adenocarcinoma [122].

These combined data layers enable the identification of distinct metabolic vulnerabilities, novel biomarkers, and potential therapeutic targets specific to NEPC. Collectively, multi-omics integration yields a systems-level portrait of NEPC biology, paving the way for precision diagnostics and subtype specific interventions, approaching the concept of personalized medicine. This approach represents a paradigm shift from traditional “one-size-fits-all” clinical trial methodologies toward precision medicine frameworks that acknowledge and leverage the molecular complexity inherent to NEPC, offering the greatest potential for meaningful therapeutic advances in this challenging disease.

As clinician-investigators, we must acknowledge that the traditional linear progression through sequential trial phases inadequately addresses the temporal urgency and molecular diversity characteristic of NEPC [123].

The treatment-emergent NED following ARPIs creates a dynamic therapeutic target that evolves during treatment, necessitating trial designs capable of real-time adaptation to changing molecular landscapes [5].

The regulatory landscape has evolved to support these innovative methodologies, with agencies recognizing the scientific and ethical imperatives for adaptive approaches in rare, aggressive malignancies [124].

Another interesting option is the use of synthetic control arms (SCAs). The application of SCAs in clinical trials represents a promising methodological innovation. SCAs leverage historical trial data or real-world evidence (RWE), such as electronic health records and registry datasets, to create a virtual comparator cohort that mirrors the experimental arm, potentially avoiding the need for concurrent randomized controls [125].

Several drugs have been developed through single-arm clinical trials and subsequently submitted to the FDA for approval based on comparisons with synthetic control arms: selumetinib, blinatumomab and others [126, 127].

This approach is particularly well-suited to NEPC, where enrolment into traditional randomized controlled trials (RCTs) is often logistically and ethically challenging given rapid disease progression and small patient numbers. Recent studies in oncology have demonstrated that SCAs derived through robust statistical matching methods (e.g., propensity scoring or Bayesian hierarchical models) can closely replicate outcomes from conventional control arms, supporting credible estimates of treatment effect. Recently also the European Medicine Agency (EMA) discussed the topic of efficacy based on single-arm trials submitted as pivotal evidence in a marketing authorization [129]. Added to this is the still limited understanding of the molecular patterns that can determine lineage plasticity: certain molecular pathways appear to play a role, yet a comprehensive vision that could prevent such an onset is still lacking. It would probably be worthwhile to ask ourselves whether it might be useful to “dust off” some therapeutic options currently considered somewhat outdated, such as developing studies aimed at testing the efficacy of intermittent regimens in cases of prolonged use of the new ARPIs. Another interesting topic is the role of the TME and immunotherapy: despite the expression of PD-L1, treatments with immune checkpoint inhibitors have led to discouraging results in t-NEPC. This, however, is in line with what has emerged in studies regarding PCas in general and arises from the lack of an advanced biomolecular classification, unlike what occurs, for example, in breast cancers [130]. However, the poor results of immunotherapy may also derive from immunosuppressive characteristics specific to PCa, such as the use of steroids during chemotherapy and abiraterone, or continuous ADT therapy.

## Conclusion

PCa is one of the tumor types that has brought the greatest scientific success over the past fifteen years, given the vast array of new therapeutic options that have significantly increased life expectancy, even for patients with metastatic disease. Within this positive framework, NEPC tumors represent the other side of the coin: this is not only due to their poor prognosis and limited treatment options, but also because the increase in cases appears paradoxically largely due to the widespread use of new hormone therapies. It is likely that other, still less-known aspects also play a significant role in this context, such as a different intestinal microbiome and/or interactions with circadian hormonal rhythms. In this context, the cutting edge of research is represented by multi-omic analyses, which could provide fundamental answers for a better understanding of t-NEPC and for the development of increasingly personalized therapy against this tumor subtype.

## Data Availability

No datasets were generated or analysed during the current study.
